# Supplementary material for: Neural signatures of indirect pathway activity during subthalamic stimulation in Parkinson’s disease
Source: Nat Commun. 2024 Apr 11;15:3130. doi: 10.1038/s41467-024-47552-6 (PMC11009243; doi:10.1038/s41467-024-47552-6)
Supplement: Supplementary file 1 — Supplementary Information [file 41467_2024_47552_MOESM1_ESM.pdf]

Supplementary Information

Supplementary Table 1: Data sources and access

Fig. 2	20 neurons / recording locations from 8 patients. For each neuron stimulation trains at three stimulation intensities included (total n = 60 recordings).<br><br>Anonymized experimental data are provided in the supplement to this publication (Var_Int_STN1 - Var_Int_STN20).<br><br>Data has been previously reported on here: https://pubmed.ncbi.nlm.nih.gov/31422369/
Fig. 3 (and Supplementary Fig. 1)	12 recording locations from 8 patients at five different stimulation frequencies (total n = 60 recordings).<br><br>Anonymized experimental data are provided in the supplement to this publication (Var_Frq_STN1 - Var_Frq_STN12).<br><br>Data has been previously reported on here: https://pubmed.ncbi.nlm.nih.gov/33991712/
Fig. 5	20 trajectories from 14 patients.<br><br>Anonymized experimental data has not been previously reported on and is provided in the supplement to this publication (ERNA_topology1 - ERNA_topology20).

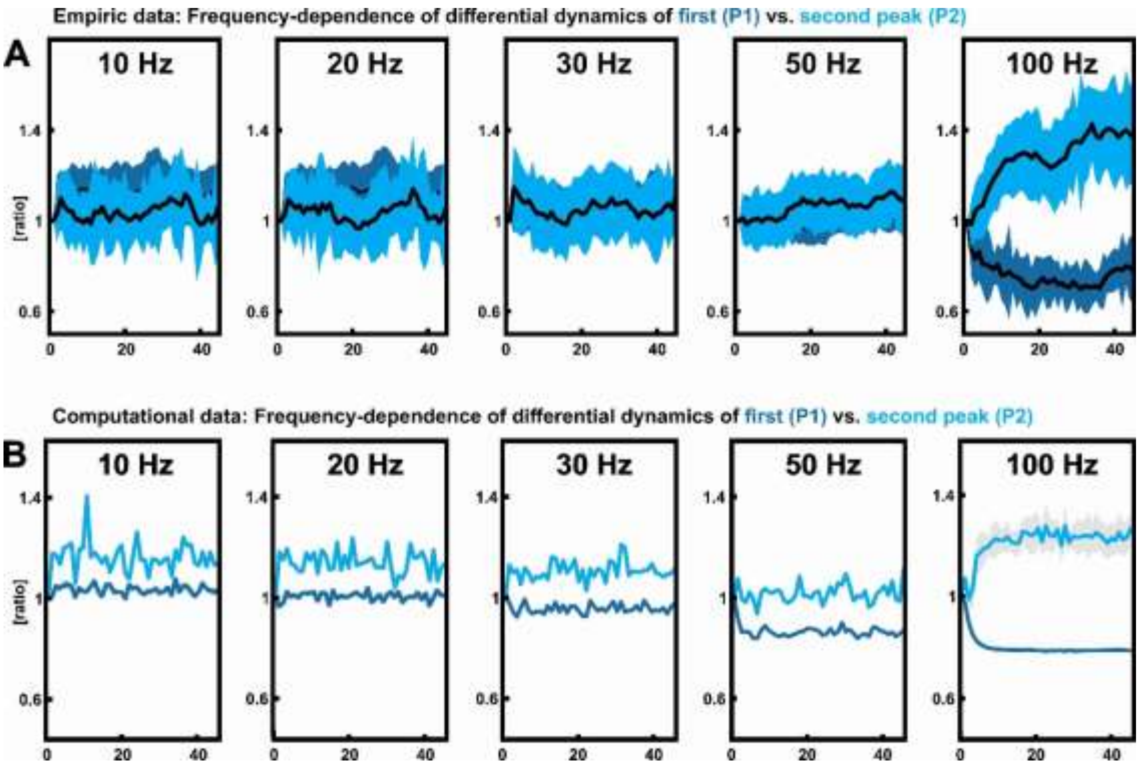

Supplementary Fig. 1 – Frequency-dependent dynamics of ERNA peaks in empiric findings and computational modelling. (A) Dynamics of first (dark blue) and second peak (light blue) taken from the same set of neurons that are shown in Fig 3 at varied stimulation frequencies, all at 100 µA. (B) Computational results for the same set of stimulation frequencies. These (A) empirical and (B) computational results suggest that the development of ERNA is dependent upon the elicitation of synapse-specific short-term synaptic dynamics achieved by high frequency stimulation.

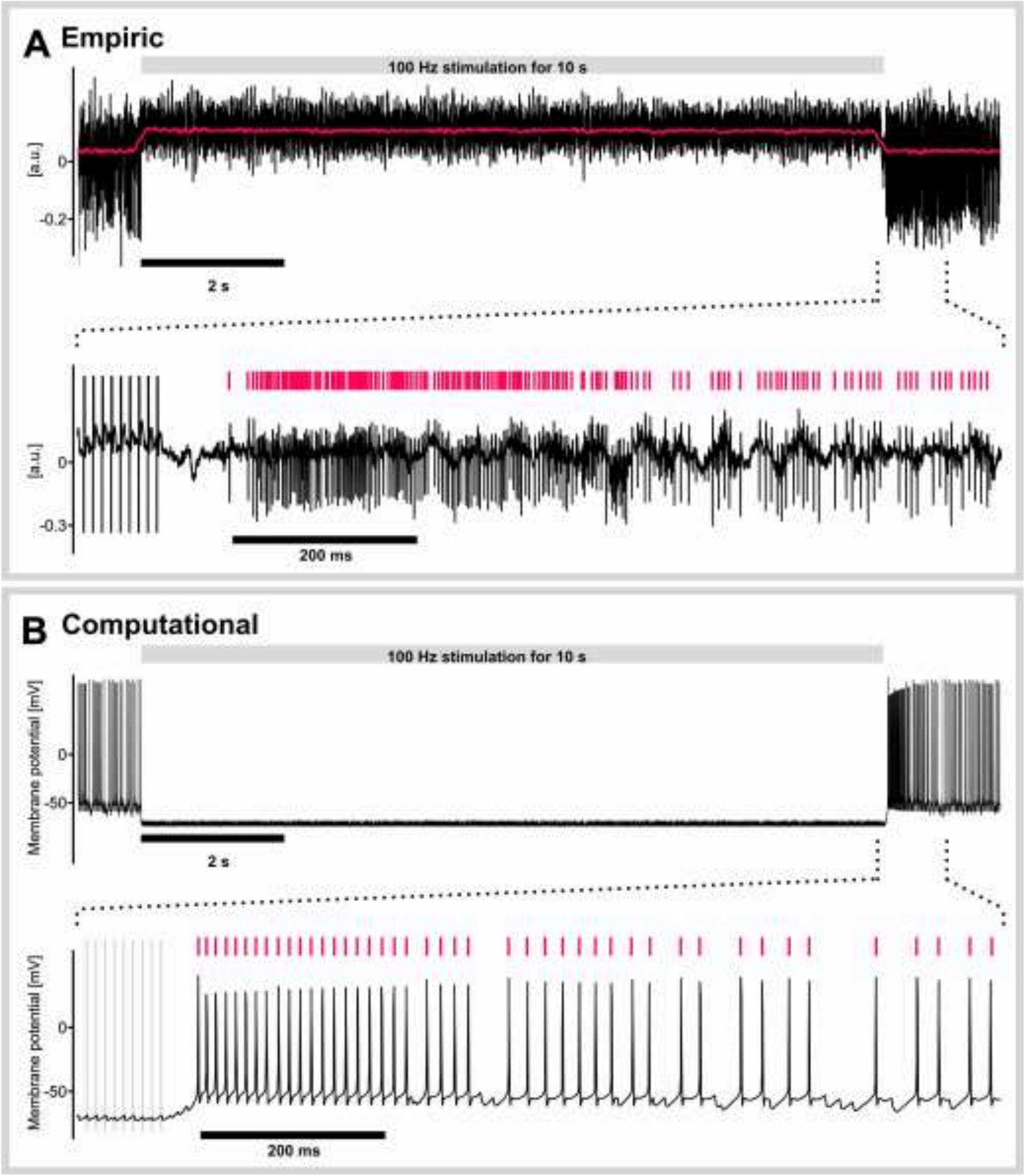

Supplementary Fig. 2 - Conductance-based model of STN confirms that spiking activity is inhibited during stimulation-induced currents and replicates rebound burst phenomenon after stimulation-induced hyperpolarization. (A) Representative example of a 10 s train of stimulation at 100 Hz (100µA; 0.3 ms biphasic pulses). Upper panel shows raw trace with stimulation artefacts removed for clarity. Note that neuronal firng is completely supressed during ongoing stimulation. Superimposed pink trace represents same recording smoothed with 0.1 s time constant to highlight net inhibitory (stimulation-induced) current produced by stimulation. (B) In silico membrane potential response of the conductance-based model of STN in response to stimulation-induced currents. (Upper panels, in A & B) 13.5 s trains of stimulation / simulation including 1s baseline firing; 10 s of stimulation / stimulation-induced currents and 1.5 s of (in silico) neuronal activity after stimulation. (Lower panels, in A & B). Same traces as in upper panels at higher temporal resolution to highlight after hyperpolarization rebound burst phenomenon in both empirical (A) and in silico data (B). Methods and Results texts related to this figure are described below.

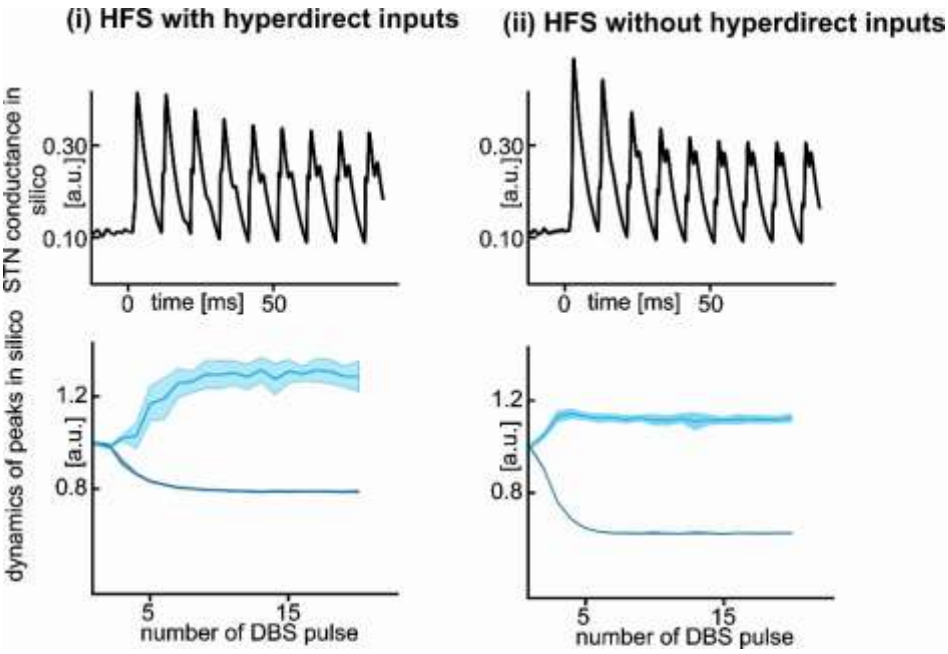

Supplementary Fig. 3 – Modeling results in the presence and absence of rapidly depressing hyperdirect inputs to STN. (Upper panels) STN conductances in response to HFS. (Lower panels) In silico dynamics of first (dark blue) and second peak (light blue) of the ERNA waveform. Note that ERNA peaks show similar dynamics in the (i) presence and (ii) absence of rapidly depressing hyperdirect inputs to STN. This suggests that hyperdirect pathway inputs are likely of negligible importance towards the generation of the ERNA waveform.

Supplementary Methods related to Fig. 2

We employed a detailed conductance model of an STN neuron (Hahn and McIntyre, Journal of computational neuroscience 2010) simulated in NEURON[1] to investigate the response characteristics during and after DBS. The STN neuron was injected with fluctuating synaptic conductances to mimic background activity so that the model had an expected baseline firing rate.[2] The conductance model was simulated receiving the synaptic activity generated from our LIF based model during DBS (i.e., *in silico* ERNA). Note that the model parameters were derived from rats[3] instead of humans, which may lead to differences in the time course of responses.

Supplementary Results related to Fig. 2

The conductance model receiving synaptic activity derived from our modelled activation of synaptic afferents (from Fig. 4 of the main manuscript) was able to produce similar post-hyperpolarization rebound bursting activity as shown in representative empiric data. During ongoing DBS, neuronal activity was completely inhibited until the stimulation train terminated, after which rebound bursting activity occurred (both *in silico* and in the representative empiric data).

References:

1. Hines, M., Davison, A. & Muller, E. NEURON and Python. *Frontiers in Neuroinformatics* **3,** (2009).

2. Destexhe, A., Rudolph, M., Fellous, J.-M. & Sejnowski, T. J. Fluctuating synaptic conductances recreate in vivo-like activity in neocortical neurons. *Neuroscience* **107,** 13–24 (2001).

3. Otsuka, T., Abe, T., Tsukagawa, T. & Song, W.-J. Conductance-Based Model of the Voltage-Dependent Generation of a Plateau Potential in Subthalamic Neurons. *Journal of Neurophysiology* **92,** 255–264 (2004).
