# Supplementary material for: Neural signatures of indirect pathway activity during subthalamic stimulation in Parkinson’s disease
Source: Nat Commun. 2024 Apr 11;15:3130. doi: 10.1038/s41467-024-47552-6 (PMC11009243; doi:10.1038/s41467-024-47552-6)
Supplement: Supplementary file 3 — Reporting Summary [file 41467_2024_47552_MOESM3_ESM.pdf]

nature portfolio

Corresponding author(s): Luka MIlosevic

Last updated by author(s): Feb 26, 2024

Reporting Summary

Nature Portfolio wishes to improve the reproducibility of the work that we publish. This form provides structure for consistency and transparency in reporting. For further information on Nature Portfolio policies, see our Editorial Policies and the Editorial Policy Checklist.

Statistics

For all statistical analyses, confirm that the following items are present in the figure legend, table legend, main text, or Methods section.

n/a	Confirmed	
☐	☒	The exact sample size (*n*) for each experimental group/condition, given as a discrete number and unit of measurement
☐	☒	A statement on whether measurements were taken from distinct samples or whether the same sample was measured repeatedly
☐	☒	The statistical test(s) used AND whether they are one- or two-sided<br>*Only common tests should be described solely by name; describe more complex techniques in the Methods section.*
☐	☒	A description of all covariates tested
☐	☒	A description of any assumptions or corrections, such as tests of normality and adjustment for multiple comparisons
☐	☒	A full description of the statistical parameters including central tendency (e.g. means) or other basic estimates (e.g. regression coefficient) AND variation (e.g. standard deviation) or associated estimates of uncertainty (e.g. confidence intervals)
☐	☒	For null hypothesis testing, the test statistic (e.g. *F*, *t*, *r*) with confidence intervals, effect sizes, degrees of freedom and *P* value noted<br>*Give P values as exact values whenever suitable.*
☒	☐	For Bayesian analysis, information on the choice of priors and Markov chain Monte Carlo settings
☒	☐	For hierarchical and complex designs, identification of the appropriate level for tests and full reporting of outcomes
☒	☐	Estimates of effect sizes (e.g. Cohen's *d*, Pearson's *r*), indicating how they were calculated

Our web collection on statistics for biologists contains articles on many of the points above.

Software and code

Policy information about availability of computer code

Data collection	Intracranial microelectrode recordings were acquired during awake DBS surgeries (at least 12h since the last dose of antiparkinsonian medication) using two closely-spaced microelectrodes (15–25 µm tips; ~600um spacing; Fig. 1).9,35 Recordings were obtained at ≥10kHz sampling frequency using two Guideline System GS3000 amplifiers (Axon Instruments, Union City, USA) and digitized using a CED1401 data acquisition system with Spike2 v7 software (Cambridge Electronic Design, Cambridge, UK). Microstimulation was delivered using a constant-current stimulator (Neuro-Amp1A, Axon Instruments, Union City, USA). All stimulation within this study was applied with 0.3 ms biphasic pulses with variable stimulation intensity, frequency, and train duration, according to the individual experiment.
Data analysis	Code availability<br>Codes for the ERNA peak extraction interface (https://github.com/Toronto-TNBS/erna),59 3D heatmap generator (https://github.com/VoodooCode14/heatmap_plotter),60 and computational modelling (https://github.com/nsbspl/ERNA-Abstract-Model)61 have been made available.

For manuscripts utilizing custom algorithms or software that are central to the research but not yet described in published literature, software must be made available to editors and reviewers. We strongly encourage code deposition in a community repository (e.g. GitHub). See the Nature Portfolio guidelines for submitting code & software for further information.

Data

Policy information about availability of data

All manuscripts must include a data availability statement. This statement should provide the following information, where applicable:

- Accession codes, unique identifiers, or web links for publicly available datasets
- A description of any restrictions on data availability
- For clinical datasets or third party data, please ensure that the statement adheres to our policy

Data availability
Experimental data (Fig. 2, Fig. 3, and Fig. 5) in this study are provided in the Supplementary Material / Source Data files; sources and access are summarized in Supplementary Table 1.

Research involving human participants, their data, or biological material

Policy information about studies with human participants or human data. See also policy information about sex, gender (identity/presentation), and sexual orientation and race, ethnicity and racism.

Reporting on sex and gender	The Canadian Institute of Health Research ("Assessing Sex and Gender Integration in Peer Review") suggests that gender-based analyses may not be possible in acute "clinical study of disease process and treatment." This is an acute electrophysiological study and the purpose is scrutinization of an electrophysiological signature.
Reporting on race, ethnicity, or other socially relevant groupings	DBS is based on clinical indications without regard to identity factors such as sex, gender, race, ethnicity, religion, sexual orientation, or socioeconomic status. Participants in our acute electrophysiological studies are those who have already elected to undergo deep brain surgery based on clinical indications.
Population characteristics	This study leveraged access to participants who already consented to undergo clinically indicated elective DBS surgery for idiopathic Parkinson's disease per standard inclusion/exclusion criteria (Munhoz RP, Picillo M, Fox SH, Bruno V, Panisset M, Honey CR, Fasano A. Eligibility criteria for deep brain stimulation in Parkinson's disease, tremor, and dystonia. Canadian Journal of Neurological Sciences. 2016 Jul;43(4):462-71.). Covariant-relevant population characteristics were not considered as this study only investigated an acute electrophysiological signatures measured during DBS surgery.
Recruitment	Participants were those who already consented to undergo clinically indicated elective DBS surgery. Those included in this work are participants who additionally agreed to undergo research-related stimulation.
Ethics oversight	University Health Network Research Ethics Board

Note that full information on the approval of the study protocol must also be provided in the manuscript.

Field-specific reporting

Please select the one below that is the best fit for your research. If you are not sure, read the appropriate sections before making your selection.

☒ Life sciences ☐ Behavioural & social sciences ☐ Ecological, evolutionary & environmental sciences

For a reference copy of the document with all sections, see nature.com/documents/nr-reporting-summary-flat.pdf

Life sciences study design

All studies must disclose on these points even when the disclosure is negative.

Sample size	We used sample sizes comparable to previous intracranial electrophysiological studies with data aquired during DBS implantation surgery (Milosevic et al., Brain Stimulation, 2021).
Data exclusions	No data were excluded from the analyses
Replication	Statistical analyses are described in depth and in a step by step manner to facilitate replication.<br>Data sample sizes vary based on individual experiment (Fig. 2: 20 unique recording locations from 8 participants; Fig. 3: 12 unique recording locations from 8 participants; Fig. 5: 20 trajectories each with multiple repeated measures; from 14 participants).<br>Data analyses and statistical analyses were performed once.
Randomization	n/a; this is not a clinical trial and results are not subject to participant or investigator bias.
Blinding	n/a; this is not a clinical trial and results are not subject to participant or investigator bias.

Reporting for specific materials, systems and methods

We require information from authors about some types of materials, experimental systems and methods used in many studies. Here, indicate whether each material, system or method listed is relevant to your study. If you are not sure if a list item applies to your research, read the appropriate section before selecting a response.

Materials & experimental systems

n/a	Involved in the study
☒	☐ Antibodies
☒	☐ Eukaryotic cell lines
☒	☐ Palaeontology and archaeology
☒	☐ Animals and other organisms
☐	☒ Clinical data
☒	☐ Dual use research of concern
☒	☐ Plants

Methods

n/a	Involved in the study
☒	☐ ChIP-seq
☒	☐ Flow cytometry
☒	☐ MRI-based neuroimaging

Clinical data

Policy information about clinical studies

All manuscripts should comply with the ICMJE guidelines for publication of clinical research and a completed CONSORT checklist must be included with all submissions.

Clinical trial registration	n/a; this is not a clinical trail.
Study protocol	n/a; this is not a clinical trail. Experimental protocols are described in depth in the methods section.
Data collection	Recordings were obtained from 30 patients with idiopathic PD during awake DBS surgeries (details provided in Supplementary Table 1). All experiments conformed to the guidelines set by the Tri-Council Policy on Ethical Conduct for Research Involving Humans and were approved by the University Health Network Research Ethics Board and each patient provided written informed consent.
Outcomes	n/a; this is not a clinical trail.

Plants

Seed stocks	*Report on the source of all seed stocks or other plant material used. If applicable, state the seed stock centre and catalogue number. If plant specimens were collected from the field, describe the collection location, date and sampling procedures.*
Novel plant genotypes	*Describe the methods by which all novel plant genotypes were produced. This includes those generated by transgenic approaches, gene editing, chemical/radiation-based mutagenesis and hybridization. For transgenic lines, describe the transformation method, the number of independent lines analyzed and the generation upon which experiments were performed. For gene-edited lines, describe the editor used, the endogenous sequence targeted for editing, the targeting guide RNA sequence (if applicable) and how the editor was applied.*
Authentication	*Describe any authentication procedures for each seed stock used or novel genotype generated. Describe any experiments used to assess the effect of a mutation and, where applicable, how potential secondary effects (e.g. second site T-DNA insertions, mosiacism, off-target gene editing) were examined.*
